# It’s Time to Dispel the Myth of “Asymptomatic” Schistosomiasis

**DOI:** 10.1371/journal.pntd.0003504

**Published:** 2015-02-19

**Authors:** Charles H. King

**Affiliations:** Center for Global Health and Diseases, Case Western Reserve University School of Medicine, Cleveland, Ohio, United States of America; Brown University, UNITED STATES

Peter Hotez and colleagues recently published a *PLOS Neglected Tropical Diseases* Viewpoint [1] on the implications of the Global Burden of Disease 2010 study (GBD 2010) for the field of neglected tropical diseases (NTDs). The article highlighted the recent improvements in the GBD’s Disability-Adjusted Life-Year (DALY) ranking system, but there remains much work to be done in reversing the DALY-mediated underestimation of NTDs’ importance to the global health burden.

With regard to the inputs used for the GBD 2010’s DALY calculations for schistosomiasis, I see a problem: the core team in charge of the GBD 2010 (the Institute for Health Metrics and Evaluation [IHME]) continues to systematically underestimate the burden of *Schistosoma* infection–related disability [2]. This underestimation is based on a flawed perception of *Schistosoma* infection and its related disease manifestations—IHME continues to adhere to the concept of “asymptomatic” schistosomiasis, while it is my considered opinion that no such health state exists.

The unfortunate use of the term “asymptomatic” implies that most *Schistosoma*-infected patients are not experiencing ongoing morbidity or disability. This is not the case because, by its nature, schistosomiasis is always a chronic inflammatory disease either of the intestine, genitourinary tract, or other organs. Eggs must cause inflammation to leave the human body to complete the parasite life cycle, and immune-mediated inflammation and scarring of the host tissues are an intrinsic part of infection [3].

“Asymptomatic schistosomiasis” was a faulty concept promulgated by Ken Warren (Rockefeller Foundation), Ken Mott (World Health Organization), and other policymakers in the 1970s and 1980s [4]. In that era, there was no affordable treatment for the millions of people who had *Schistosoma*-associated disease. Apparently, in that context, policymakers’ cognitive filters allowed them to accept the idea of a benign, “asymptomatic” form of human *Schistosoma* infection that could go untreated. However, this construct was not supported by the data. It grew out of a misinterpretation of Warren’s population-based field surveys of schistosomiasis [5,6]. Those studies used Kato-Katz stool smears and urine filtration egg count data to show that patients with higher egg counts had greater risk of symptoms and objective morbidity in *Schistosoma*-endemic areas. On that, we can agree. Nevertheless, the published data indicated that both subjects with “light” infections and those with no eggs detected (“endemic controls”) had appreciable rates of symptoms and morbidity, as well.

Warren’s conclusion seems to have been that there was no association between light infection and disease, because the “light infection” group was not significantly different from those who were “non-infected” (albeit, based only on limited testing) within the same area. Actually, the presence of a significant number of light infections in the “non-infected” control group likely led to a dilution in the observed differences between study groups. His interpretation afforded the conclusion that people with light infections must be relatively “asymptomatic” from their *Schistosoma* infections.

But this misinterpretation was based on a diagnostic misclassification bias created by the insensitivity of single-specimen stool or urine egg count testing for infection. Most likely, many (at least 20%–40%) of his egg-negative subjects were actively infected [7–9] and had symptoms and pathology from their *Schistosoma* infection. Therefore, his comparisons between “infected” and “uninfected,” and between heavily infected and “uninfected” subjects, were flawed. Leading schistosomiasis experts, including Warren [9,10], knew of the insensitivity of egg count diagnostics but apparently chose to ignore this important methodological issue. In terms of formulating 1980s policy for schistosomiasis control, given the lack of affordable treatments without significant side effects, it is likely that low intensity infections were not prioritized and even dismissed. In that era, the strategy promoted for schistosomiasis control was to focus very limited treatment resources on treatment and prevention of heavy *Schistosoma* infections [11,12]. The initial GBD 1996 DALY rankings for schistosomiasis (which immediately followed that era) mirror this earlier bias, as reflected in Mott’s synopsis of schistosomiasis for the initial GBD 1996 program [4]. The difficulty of accurate case-classification persists into the 2001 van der Werf et al. reviews [13] that IHME has used as part of the newer GBD 2010 estimations of schistosomiasis burden.

In his 1980 review regarding prospects for *Schistosoma* control [11], Peter Jordan, Director of the multiyear Rockefeller-funded schistosomiasis control project in St. Lucia, agreed that the focus for “disease control” should be the prevention of heavy infections, but he admitted that “…‘measures of health’ are as yet insensitive and cannot measure the ill health caused by low numbers of trematodes…” In 2014, we have much more extensive knowledge of the links between past or present *Schistosoma* infection, per se, and the “subtle” morbidities of pain, dysuria, dyspareunia, fatigue, anemia, growth stunting, undernutrition, cognitive impairment, and genital disease [14,15]. While these morbidities may not be overtly symptomatic in terms of creating immediate demand for clinical care, they are undoubtedly physically and socially disabling for most affected patients. Moreover, a patient can continue to have the disease schistosomiasis even after *Schistosoma* infection is ended. Included in this latter category is the risk of cancer caused by years of chronic tissue inflammation.

So, it is clear that in accounting *Schistosoma*-related disease burden, we need to include all “egg-negative schistosomiasis” detected by newer disease classifications. While the GBD 2010 now accounts for some milder symptoms (diarrhea, dysuria, anemia) of active schistosomiasis, it does not accord them much disability. It includes separate accounting of advanced forms of disease (hepatic inflammation, hematemesis, and ascites), but it does not include advanced urogenital diseases, infertility, or the late effects of growth stunting and cognitive impairment. Including these disease outcomes could effectively double the number of people considered to be affected by significant *Schistosoma*-related disease.

Historically, research to specify these impacts has been hampered by poor diagnostics and scarce funding. It is likely, given the now-evident risks of leaving *Schistosoma* infection untreated, that any future placebo-controlled trials will be considered to be unethical to perform. As a result, we may never have a perfectly clear picture of the attributable risk for all the morbid conditions associated with *Schistosoma* infection. I am fairly convinced, however, that very few people living in a high-risk, *Schistosoma*-endemic area escape infection, and I believe that all those who are infected are symptomatic or diseased to some extent. Current mass drug administration programs, in which praziquantel therapy is given irrespective of individuals’ “egg-positive” or “egg-negative” status, are now the interventions most likely to unmask the true impact of chronic *Schistosoma* infection.

In brief, patients with chronic intestinal or bladder pathology cannot be “asymptomatic,” as imputed by the GBD 2010 disability weights assigned to the majority of *Schistosoma*-infected patients [2]. From my viewpoint, there is no benign, “asymptomatic” form of schistosomiasis that can be dismissed with vanishingly small disability weights. Praziquantel treatment is now cheap and widely available. It is time to quit believing in the myth of “asymptomatic schistosomiasis” and account for the disease as it really is, so that it can be rightly controlled and prevented.
